# Antibodies to mutated citrullinated vimentin and disease activity score in early arthritis: a cohort study

**DOI:** 10.1186/ar2362

**Published:** 2008-01-28

**Authors:** Jennie Ursum, Markus MJ Nielen, Dirkjan van Schaardenburg, Ann R van der Horst, Rob J van de Stadt, Ben AC Dijkmans, Dörte Hamann

**Affiliations:** 1 Jan van Breemen Institute, Dr Jan van Breemenstraat, 1056 AB Amsterdam, The Netherlands; 2 VU University Medical Centre, 1007 MB Amsterdam, The Netherlands; 3 Sanquin Diagnostic Services, 1006 AD Amsterdam, The Netherlands

## Abstract

**Introduction:**

The aim of our study was to investigate the association between arthritic disease activity and antibodies to mutated citrullinated vimentin (anti-MCV), because such a relation has been suggested.

**Methods:**

Anti-MCV levels were measured in 162 patients with early arthritis (123 with rheumatoid arthritis and 39 with undifferentiated arthritis) at baseline and at 1 and 2 years of follow up. Disease activity was measured using the disease activity score (Disease Activity Score based on 28 joints [DAS28]) and serum C-reactive protein. General estimation equation analysis was used to assess the relation between anti-MCV levels and DAS28 over time.

**Results:**

Both, anti-MCV levels and DAS28 exhibited a significant decrease during the first and second year. However, the association between anti-MCV levels and DAS28, adjusted for dependency on sequential measurements within one individual, was very low (β = 0.00075). In a population of patients with rheumatoid arthritis or undifferentiated arthritis, anti-MCV had a specificity of 92.3% and a sensitivity of 59.3% when using the recommended cut-off of 20 U/ml. Specificity and sensitivity of antibodies against second-generation cyclic citrullinated peptide, using the recommended cut-off value of 25 U/ml, were 92.1% and 55.3%, respectively. Anti-MCV-positive early arthritis patients had significantly higher Sharp-van der Heijde score, erythrocyte sedimentation rate and C-reactive protein levels than did anti-MCV-negative patients at all time points (*P *< 0.005), but DAS28 was higher in anti-MCV-positive patients at 2 years of follow up only (*P *< 0.05).

**Conclusion:**

Because the correlation between anti-MCV levels and parameters of disease activity was very low, we conclude that it is not useful to monitor disease activity with anti-MCV levels.

## Introduction

At present, two types of serological markers are used in the early diagnosis of rheumatoid arthritis (RA): antibodies to the Fc part of human IgG (rheumatoid factor) and antibodies to citrullinated protein/peptide antigens (ACPAs). Rheumatoid factor is not specific to RA, because it is present in patients suffering from other autoimmune and/or infectious diseases and are found in apparently healthy elderly patients [1]. ACPAs have a high specificity for RA. Since the first description of RA-specific antibodies to citrullinated peptides, several citrullinated proteins have been proposed as physiological targets for ACPA specificity, such as fibrin [2], Epstein-Bar virus nuclear antigen [3], α-enolase [4] and vimentin [5].

Antibodies against second-generation cyclic citrullinated peptide (anti-CCP2) are frequently used by clinicians to assess RA. Anti-CCP2 level has a good diagnostic and prognostic value [6], but in one study [7] no relation between anti-CCP2 levels and a disease activity score (Disease Activity Score based on 28 joints [DAS28]) was identified. Citrullinated vimentin has been shown to be the target for the previously described RA-specific Sa antibodies [5]. In a cohort of patients with early arthritis, the presence of Sa antibodies correlated with a more dramatic disease presentation [8]. Recently, an ELISA for the detection of antibodies against human mutated citrullinated vimentin (MCV) was developed [9]. Anti-MCV level had high sensitivity in patients with established RA [9,10]. Also, anti-MCV levels correlated with DAS28 score in a small population of 21 patients with RA over a period of 3 years [9]. The present study focuses on the association between anti-MCV levels and DAS28 in patients with early arthritis over 2 years of follow up. In addition, sensitivity and specificity of the anti-MCV test were determined in a group of patients with early arthritis.

## Materials and methods

### Patients

The study population included 162 patients (age ≥ 18 years) with peripheral arthritis of two or more joints and with symptom duration of 3 years or less, who had been newly referred to the early arthritis clinic of the Jan van Breemen Institute (a large rheumatology clinic in Amsterdam, The Netherlands) between 1995 and 1998. Patients who were previously treated with a disease-modifying antirheumatic drug and patients with spondylarthropathy, reactive arthritis, crystal-induced arthropathy, systemic lupus erythematosus, Sjögren's syndrome, or osteoarthritis were excluded. Baseline was defined as the first presentation to the rheumatologist. Sera were available from baseline and at 1 and 2 years of follow up. After 1 year of follow up patients were diagnosed as having RA or undifferentiated arthritis (UA) after chart review by an experienced rheumatologist (BD), who was blinded to the results of antibody testing. The local ethics committee (Slotervaart Hospital, Jan van Breemen Institute and BovenIJ Hospital, Amsterdam, The Netherlands) approved the study protocol. All patients gave written informed consent to be included in the study.

### Disease parameters

At baseline the following data were collected: demographic characteristics; disease duration; disease activity, as measured using DAS28 [11]; patient pain, using visual-analogue scale (VAS); and functional status, causing Health Assessment Questionnaire [12]. Laboratory assessments at baseline included erythrocyte sedimentation rate (ESR), C-reactive protein (CRP), and anti-CCP2. Radiographs of hands and feet were obtained at baseline, and at 1 and 2 years. The number of erosions and the joint space narrowing were scored according to the Sharp/van der Heijde method [13] by an experienced rheumatologist (DvS), who was unaware of the other data.

### Antibody measurements

Anti-CCP2 levels were measured using the second-generation Immunoscan RA ELISA kit (Euro-diagnostica, Arnhem, The Netherlands) with a cut-off value of 25 U/ml, as proposed by the manufacturer. ELISA kits for detection of anti-MCV were generously provided by ORGENTEC Diagnostica GmbH (Mainz, Germany) and were used in accordance with the manufacturer's instructions [9] with the recommended cut-off value of 20 U/ml. Anti-CCP2 levels were determined at baseline and anti-MCV levels were measured at baseline and at 1 and 2 years of follow up.

### Analysis

The baseline characteristics of the RA patients and UA patients were compared using Student's *t*-test, the Mann-Whitney U-test and the χ^2 ^test, as appropriate. Correlation between anti-MCV levels and DAS28, anti-CCP2 levels, or radiographic progression was determined using Spearman correlation. General estimation equation (GEE) analysis was used to determine the relation between anti-MCV levels and DAS28 over time. This regression technique was used because it adjusts for dependency of several measurements within one individual and it is capable of dealing with missing data [14]. Sensitivity, specificity and positive predictive value of anti-MCV were calculated in the 162 patients with early arthritis.

Sensitivity expresses the percentage of RA patients who was positive for the test, whereas specificity is calculated from the percentage of test-negative UA patients.

Radiographic progression was defined as an increase of Sharp-van der Heijde score (SHS) of at least 5 after 2 years of follow up [15]; the remaining patients were classified as being nonprogressive. GEE analysis was performed using STATA 7.0 (Stata Corp., College Station, TX, USA); other statistical analyses were performed using SPSS 15.0 software (SPSS Institute Inc., Cary, NC, USA).

## Results

### Relationship of serum anti-MCV levels with the course of disease activity

The median anti-MCV level at baseline was 14 U/ml (interquartile range 6 to 289 U/ml) and at 1 year and 2 years of follow up the levels were 9 U/ml (5 to 197 U/ml) and 10 U/ml (4 to 153 U/ml), respectively. Anti-MCV values were missing in two UA patients at 1 year of follow up and in one RA patient at 2 years of follow up. Anti-MCV levels as well as DAS28 decreased significantly during the first year, but changes in DAS28 did not correlate with changes in anti-MCV levels (*r *= 0.06; *P *= 0.44). The correlations between anti-MCV level and CRP at all time points combined (*r *= 0.23; *P *< 0.01) and between anti-MCV level and DAS28 at all time points combined (*r *= 0.17; *P *< 0.05) were rather low. The correlations between anti-MCV level and two separate components of DAS28 (namely, ESR and swollen joint count [of a total of 28 joints]) were also rather low (*r *= 0.27 and *r *= 0.11, respectively; *P *< 0.05). No correlation was identified between tender joint count (of a total of 28 joints) and general health determined using a VAS (*r *= 0.2 and *r *= 0.03, respectively; *P *> 0.05). Correlations between anti-MCV levels at baseline and SHS at 2 years of follow up and between anti-CCP2 levels and SHS at 2 years of follow up were similar (*r *= 0.42 and *r *= 0.41, respectively; *P *< 0.01). No correlation was found between anti-CCP2 levels and DAS28 at baseline (*r *= 0.12; *P *= 0.14).

GEE analysis revealed a significant association between DAS28 and anti-MCV during 2 years of follow up, although the association was very low. The β coefficient (slope of the regression) of anti-MCV was small (β = 0.00075; *P *< 0.0001). Thus, if anti-MCV levels increase by 1 unit, DAS28 score will rise 0.00075 points. The β can be multiplied by the increase in anti-MCV levels to calculate the increase in DAS28. To study the relationship between anti-MCV levels and DAS28 in more detail, patients were categorized as being positive or negative for anti-MCV at baseline. Of the 162 patients in our cohort, 76 (73 patients with RA and three patients with UA) were positive for anti-MCV at baseline. The mean age, percentage of females and mean disease duration were not different between the two groups. A few patients changed anti-MCV status in time (10/162 [6.2%]). Two initially anti-MCV-negative RA patients became positive, although their anti-MCV levels were just above the cut-off point. Eight initially anti-MCV-positive patients became negative (one UA patient and seven RA patients). Anti-MCV levels from these patients were low at baseline (20 to 119 U/ml).

**Figure 1:**
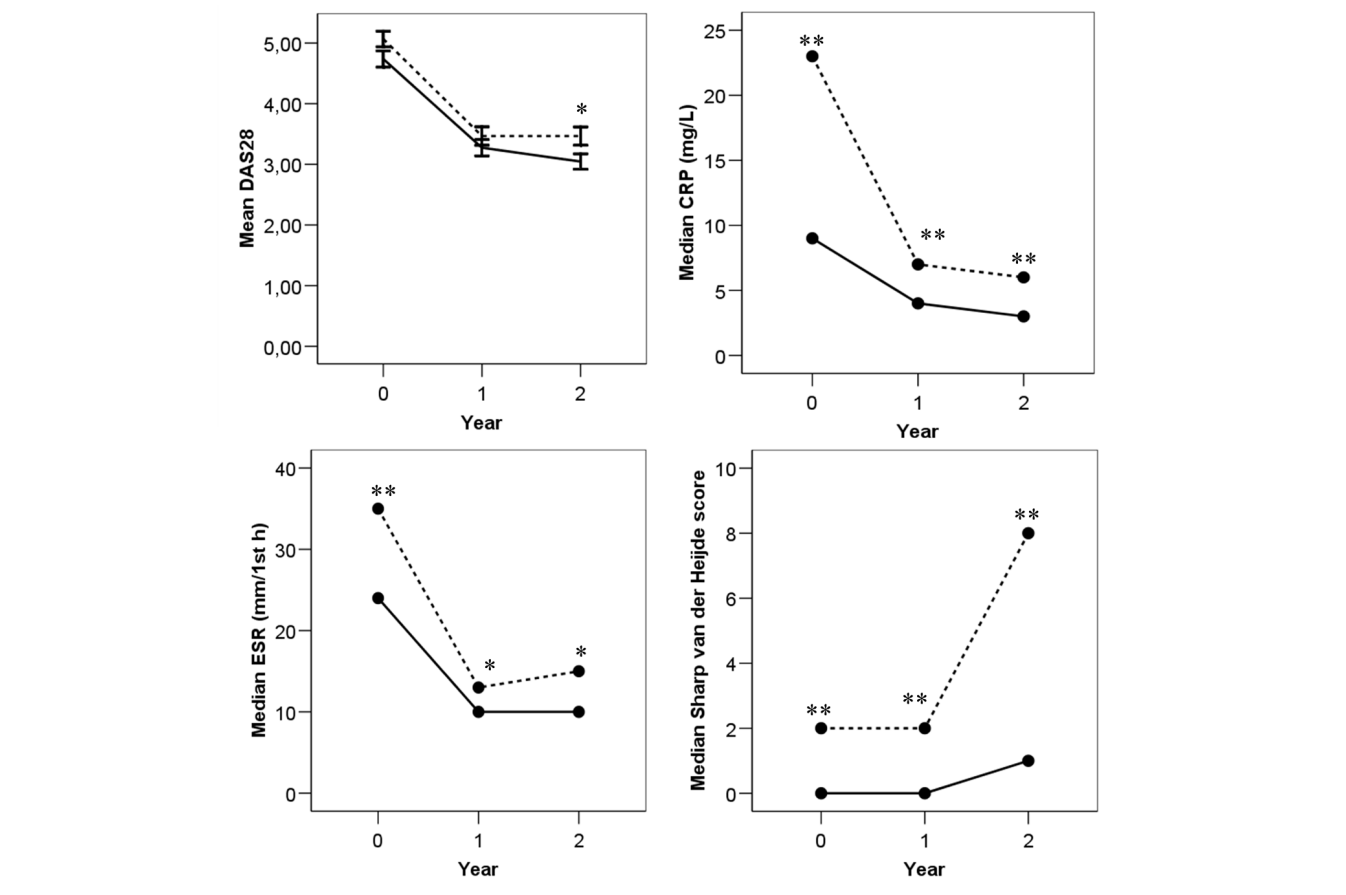
Arthritic disease activity: anti-MCV positive versus anti-MCV negative patients. Statistical comparisons were performed between anti-mutated citrullinated vimentin (anti-MCV)-positive and anti-MCV-negative patients at the indicated time points. Dotted lines represent anti-MCV-positive patients, and straight lines represents anti-MCV-negative patients. *P < 0.05; **P < 0.005. CRP, C-reactive protein; DAS28, Disease Activity Score based on 28 joints; ESR, erythrocyte sedimentation rate.

In the anti-MCV-positive and anti-MCV-negative patient group, mean DAS28 score decreased mainly during the first year, from 5.0 and 4.8 to 3.5 and 3.3, respectively. During the second year, mean DAS28 score of anti-MCV-positive patients remained stable, whereas mean DAS28 score of anti-MCV-negative patients decreased further (mean DAS28 scores 3.5 and 3.1, respectively). At baseline, DAS28 tended to be higher in anti-MCV-positive patients (*P *= 0.08). The difference became significant after 2 years of follow up (*P *< 0.05; Figure 1). Anti-MCV-positive patients had higher median ESR and CRP levels (*P *< 0.05 and *P *< 0.005, respectively) than did anti-MCV-negative patients at all time points (Figure 1). In addition to differences in parameters of inflammation, significant differences in radiographic damage were also found (Figure 1). Anti-MCV-positive patients had more radiographic damage at all time points (*P *< 0.005). There was no correlation between symptom duration and degree of radiographic damage at baseline (*r *= 0.06, *P *= 0.47). The mean duration of symptoms was not different between anti-MCV-positive and anti-MCV-negative patients (0.59 and 0.60 years, respectively).

In addition, a comparison between anti-MCV-positive and anti-MCV-negative RA patients was conducted, because the main group of UA patients appeared to be anti-MCV negative. Except for the median SHS at baseline (*P *= 0.002), there were no differences between the groups of anti-MCV-positive and anti-MCV-negative RA patients at baseline. DAS28 score did not differ significantly between the two groups over 2 years of follow up. Anti-MCV-positive RA patients had higher median CRP levels at 1 and 2 years of follow up and higher median ESR levels at 2 years of follow up (all *P *< 0.05) than did anti-MCV-negative RA patients. In addition, anti-MCV-positive RA patients had more radiographic damage at all time points (*P *< 0.005).

### Value of the anti-MCV test in early arthritis

At baseline, RA patients had a higher median ESR, CRP and SHS, and a higher mean DAS28 score and Health Assessment Questionnaire score than did UA patients (Table 1). Sensitivity, specificity and positive predictive value of anti-MCV for the diagnosis of RA at 1 year of follow up are shown in Table 2. Positivity for anti-MCV and anti-CCP was largely overlapping, although six of the 90 anti-CCP2-negative patients were positive for anti-MCV at baseline. Five of them had been diagnosed with RA and one with UA by an experienced rheumatologist who was blinded to the ACPA findings after the first year. One patient, diagnosed with UA, was positive for anti-CCP but negative for anti-MCV. Levels of anti-MCV and anti-CCP2 at baseline were highly correlated (*r *= 0.85; *P *< 0.01). The percentage of patients who used methotrexate during 2 years follow up was significantly higher in the anti-MCV-positive group than in the anti-MCV-negative group (43% versus 28%; *P *= 0.039). In addition, the percentage of patients who used sulfasalazine was significantly higher in the anti-MCV-positive group than in the anti-MCV-negative group (75% versus 60%; *P *= 0.049).

**Table 1:** Baseline characteristics of the early arthritis population: RA and UA patients

Baseline characteristics	RA (*n *= 123)	UA (*n *= 39)	*P *value
Age (years)^a^	56.4 ± 15.0	53.8 ± 14.0	NS
Female (%)^b^	86 (69.9)	26 (66.7)	NS
Disease duration (years)^c^	0.4 (0.3 to 0.6)	0.4 (0.3 to 0.8)	NS
ESR (mm/hour)^a^	36.7 ± 24.4	23.7 ± 21.0	<0.005
CRP (mg/dl)^c^	19.5 (6.5 to 45.5)	6 (2 to 15)	<0.005
Anti-CCP2 positive (%)	68 (55.3)	3 (7.9)	<0.005
Anti-MCV positive (%)	73 (59.3)	3 (7.7)	<0.005
DAS28 score^a^	5.1 ± 1.2	4.3 ± 1.2	<0.01
Sharp-van der Heijde score^c^	2 (0 to 6)	0 (0 to 1)	<0.01
HAQ score^a^	1.1 ± 0.7	0.8 ± 0.6	p < 0.05

Data are expressed as mean ± standard deviation or as median (Interquartile range). ^a^Tested using Student's *t*-test. ^b^Tested using the χ^2 ^test. ^c^Tested using the Mann Whitney U-test. CCP2, second-generation cyclic citrullinated peptide; CRP, C-reactive protein; DAS28, Disease Activity Score of 28 joints; ESR, erythrocyte sedimentation rate; HAQ, Health Assessment Questionnaire; MCV, mutated citrullinated vimentin; NS, not significant; RA, rheumatoid arthritis; UA, undifferentiated arthritis.

**Table 2:** Sensitivity, specificity and positive predictive value of anti-MCV and anti-CCP for clinical diagnosis of RA in early arthritis patients

Antibody	Sensitivity (%; 95% CI)	Specificity (%; 95% CI)	PPV (%)
Anti-CCP2 level ≥ 25 U/ml	55.3 (46.1 to 64.2)	92.1 (77.5 to 97.9)	95.8
Anti-MCV level ≥ 20 U/ml	59.3 (50.1 to 67.9)	92.3 (78.0 to 97.9)	96.1

Shown are the sensitivity, specificity and positive predictive value (PPV) of anti-mutated citrullinated vimentin (anti-MCV) and anti-second-generation cyclic citrullinated peptide (anti-CCP2) for the clinical diagnosis of rheumatoid arthritis (RA) in early arthritis, using cut off values recommended by the manufacturers of the tests. A total of 162 patient with early arthritis were included in the analysis. CI, confidence interval.

## Discussion

The main focus of our study was to investigate the relation between anti-MCV levels and disease activity. Previously, Bang and coworkers [9] analyzed consecutive samples of 21 patients with established RA over a 2-year period of follow up and identified a correlation between anti-MCV levels and DAS28 score (Pearson correlation coefficient 0.404), whereas no correlation was found between anti-CCP2 levels and DAS28 score. Although in our cohort of 76 anti-MCV-positive patients with early arthritis the median anti-MCV level and DAS28 score both decreased over time, GEE analysis revealed a rather small association between anti-MCV levels and DAS28 score over 2 years of follow up. This implies that a large decline in anti-MCV level is needed to yield a substantial decrease in DAS28. A minimal reduction of 0.6 points in DAS28 is necessary for a moderate response, according to the European League Against Rheumatism response criteria [16]. Anti-MCV levels would have to decrease by 800 U/ml to account for such a reduction in DAS28 score. In our cohort, only five out of the 162 patients had a change of at least 800 U/ml during the first year and one patient in the second year. In addition, we did not identify a direct relationship between changes in anti-MCV levels and changes in disease activity, expressed as DAS28 score. The correlation between anti-MCV levels and CRP, DAS28, or its separate components ESR and swollen joint count was rather low, and was absent between anti-MCV levels and tender joint count and VAS general health. Previous studies of anti-CCP2 levels and disease activity yielded inconsistent results. One study [17] found corresponding changes between these two parameters, whereas others found no correlation [7,18]. In our cohort we did not find a substantial association between anti-MCV levels and disease activity. Together with the high correlation between anti-MCV and anti-CCP2 levels, our data suggest that testing ACPA on citrullinated protein substrates is comparable to testing on artificial citrullinated peptides, and that anti-MCV levels cannot be used to monitor disease activity directly.

When comparing patients with established RA versus control patients, reported sensitivities and specificities of anti-MCV ranged from 69.5% to 74.5% versus 90.3% to 93.1%, respectively [10,19,20]. These studies also used the recommended cut-off value of 20 U/ml. In our early arthritis population comprising only RA and UA patients, the sensitivity of 59.3% was lower than previously reported sensitivities. The specificity of 92.3% was rather low but comparable to those of previous studies. Interestingly, the specificity of anti-CCP2 using a cut-off value of 25 U/ml was also low (92.1%) and comparable to that of anti-MCV in our cohort. The low specificities for both ACPA tests might be explained by the inclusion of RA and UA patients only. It cannot be excluded that UA patients will convert to RA later in time. Thus far (10 years from inclusion), five of the UA patients have developed RA. One out of three anti-MCV-positive patients and four out of 36 anti-MCV-negative patients developed RA. Taking that into account, the sensitivity of anti-MCV will slightly decrease and specificity will slightly increase.

Remarkably, at baseline anti-MCV-positive RA patients already had significantly greater radiologic damage than did anti-MCV-negative RA patients. This could not be accounted for by longer symptom duration in anti-MCV-positive patients. Clinicians were unaware of anti-CCP2 or anti-MCV status because these were determined retrospectively and patients were treated according to DAS28 score. Nevertheless, despite this treatment the difference in radiological damage became even more evident after 2 years of follow up. Differences in disease activity have been reported for anti-CCP2-positive patients with early arthritis after the first [7] and second year [21] of follow up. In our cohort, at baseline anti-CCP2-positive patients were similar to anti-MCV-positive patients, and exhibited significantly higher levels of CRP and ESR and a higher SHS.

## Conclusion

In summary, anti-MCV-positive patients exhibited higher levels of inflammation and more radiographic damage at baseline than did anti-MCV-negative patients. Both DAS28 and anti-MCV levels decreased significantly during 2 years of follow up. However, because the correlation between anti-MCV levels and parameters of disease activity was very low, it is not useful to measure anti-MCV levels to monitor disease activity.

## Abbreviations

ACPA = antibody to citrullinated protein/peptide antigens; CCP2 = second-generation cyclic citrullinated peptide; CRP = C-reactive protein; DAS28 = Disease Activity Score based on 28 joints; ELISA = enzyme-linked immunosorbent assay; ESR = erythrocyte sedimentation rate; GEE = general estimation equation; MCV = mutated citrullinated vimentin; RA = rheumatoid arthritis; SHS = Sharp-van der Heijde score; UA = undifferentiated arthritis; VAS = visual-analogue scale.

## Competing interests

The authors declare that they have no competing interests.

## Authors' contributions

JU performed statistical analyses, interpreted the data and drafted the manuscript. MJN contributed to the study design and statistical analysis. DvS helped to draft the manuscript. ARvdH performed laboratory work. RJvdS provided patient material. BD was involved in classifying the clinical diagnosis. DH participated in study design, interpreted the data and drafted the manuscript.
